# Effect of chronic mucus hypersecretion on treatment responses to inhaled therapies in patients with chronic obstructive pulmonary disease: Post hoc analysis of the IMPACT trial

**DOI:** 10.1111/resp.14339

**Published:** 2022-08-15

**Authors:** Philip J. Thompson, Gerard J. Criner, Mark T. Dransfield, David M. G. Halpin, MeiLan K. Han, David A. Lipson, Ghassan J. Maghzal, Fernando J. Martinez, Dawn Midwinter, Dave Singh, Lee Tombs, Robert A. Wise

**Affiliations:** ^1^ The Lung Health Clinic Nedlands Western Australia Australia; ^2^ Lewis Katz School of Medicine at Temple University Philadelphia Pennsylvania USA; ^3^ Division of Pulmonary, Allergy, and Critical Care Medicine, Lung Health Center University of Alabama at Birmingham Birmingham Alabama USA; ^4^ University of Exeter Medical School, College of Medicine and Health University of Exeter Exeter UK; ^5^ Pulmonary & Critical Care University of Michigan Ann Arbor Michigan USA; ^6^ GlaxoSmithKline Collegeville Pennsylvania USA; ^7^ Perelman School of Medicine University of Pennsylvania Philadelphia Pennsylvania USA; ^8^ GSK Melbourne Victoria Australia; ^9^ Weill Cornell Medicine New York New York USA; ^10^ GSK Brentford UK; ^11^ Centre for Respiratory Medicine and Allergy, Institute of Inflammation and Repair, Manchester Academic Health Science Centre The University of Manchester, Manchester University NHS Foundation Hospital Trust Manchester UK; ^12^ Precise Approach Limited London UK; ^13^ Division of Pulmonary and Critical Care Medicine Johns Hopkins University School of Medicine Baltimore Maryland USA

**Keywords:** chronic mucus hypersecretion, chronic obstructive pulmonary disease, clinical outcomes, COPD, single‐inhaler triple therapy

## Abstract

**Background and objective:**

Chronic mucus hypersecretion (CMH) is a clinical phenotype of COPD. This exploratory post hoc analysis assessed relationship between CMH status and treatment response in IMPACT.

**Methods:**

Patients were randomized to once‐daily fluticasone furoate/umeclidinium/vilanterol (FF/UMEC/VI) 100/62.5/25 μg, FF/VI 100/25 μg or UMEC/VI 62.5/25 μg and designated CMH+ if they scored 1/2 in St George's Respiratory Questionnaire (SGRQ) questions 1 and 2. Endpoints assessed by baseline CMH status included on‐treatment exacerbation rates, change from baseline in trough forced expiratory volume in 1 second, SGRQ total score, COPD Assessment Test (CAT) score, proportion of SGRQ and CAT responders at Week 52 and safety.

**Results:**

Of 10,355 patients in the intent‐to‐treat population, 10,250 reported baseline SGRQ data (CMH+: 62% [*n* = 6383]). FF/UMEC/VI significantly (*p* < 0.001) reduced on‐treatment moderate/severe exacerbation rates versus FF/VI and UMEC/VI in CMH+ (rate ratio: 0.87 and 0.72) and CMH− patients (0.82 and 0.80). FF/UMEC/VI significantly (*p* < 0.05) reduced on‐treatment severe exacerbation rates versus UMEC/VI in CMH+ (0.62) and CMH− (0.74) subgroups. Similar improvements in health status and lung function with FF/UMEC/VI were observed, regardless of CMH status. In CMH+ patients, FF/VI significantly (*p* < 0.001) reduced on‐treatment moderate/severe and severe exacerbation rates versus UMEC/VI (0.83 and 0.70).

**Conclusion:**

FF/UMEC/VI had a favourable benefit: risk profile versus dual therapies irrespective of CMH status. The presence of CMH did not influence treatment response or exacerbations, lung function and/or health status. However, CMH did generate differences when dual therapies were compared and the impact of CMH should be considered in future trial design.

## Graphical abstract

The relationship between chronic mucus hypersecretion (CMH) and treatment response to FF/UMEC/VI, FF/VI or UMEC/VI was assessed in symptomatic patients with COPD and a history of exacerbations. FF/UMEC/VI reduced exacerbation rates versus FF/VI and UMEC/VI irrespective of CMH status; similar improvements in health status and lung function were observed.

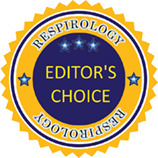

**See related** Editorial

## INTRODUCTION

Chronic obstructive pulmonary disease (COPD) is a heterogeneous disease with several clinical phenotypes, including emphysema and chronic mucus hypersecretion (CMH), which is a symptom of chronic bronchitis.^1^, ^2^, ^3^, ^4^ CMH is defined by a medical history of chronic cough and sputum,^1^, ^2^, ^5^ and characterized by an increase in goblet cells, enlarged submucosal glands and mucus production, leading to airway obstruction and productive cough.^1^, ^4^ Patients with CMH are more likely to have a heavier burden of bacterial colonization of airways, more frequent and more severe exacerbations and reduced lung function and health status, compared with patients without CMH.^1^, ^2^, ^3^, ^4^, ^6^, ^7^ It is not known whether the clinical features of CMH affect response to COPD inhaled treatment. An understanding of the impact of CMH in this regard may help predict treatment response, achieve a more personalized approach to COPD treatment and potentially lead to development of new therapies.^2^

The IMPACT trial found that once‐daily fluticasone furoate/umeclidinium/vilanterol (FF/UMEC/VI) reduced moderate/severe exacerbation rates and improved lung function and health status versus FF/VI and UMEC/VI, with a similar safety profile, in patients with symptomatic COPD at risk of exacerbations.^8^ This exploratory post hoc analysis of the IMPACT trial evaluated CMH prevalence in the trial population and assessed the effect of baseline CMH status on treatment response.

## METHODS

### Study design

The IMPACT trial (GSK CTT116855/NCT02164513) was a 52‐week, Phase III, multicentre, randomized, double‐blind and parallel‐group study.^8^, ^9^ The trial design has previously been published.^8^, ^9^ Briefly, following an open‐label, 2‐week run‐in period on existing COPD medications, patients were randomized (2:2:1) to once‐daily FF/UMEC/VI 100/62.5/25 μg, FF/VI 100/25 μg or UMEC/VI 62.5/25 μg.^8^, ^9^

### Study population

Full eligibility criteria for the IMPACT trial have been described previously.^8^, ^9^ Eligible patients were ≥ 40 years of age, had a COPD Assessment Test (CAT) score ≥ 10 and either a post‐bronchodilator forced expiratory volume in 1 second (FEV_1_) <50% predicted and a history of ≥1 moderate/severe exacerbation, or a post‐bronchodilator FEV_1_ 50%–<80% predicted and ≥2 moderate or ≥1 severe exacerbation in the previous 12 months. Patients with a concomitant diagnosis of asthma or other respiratory disorders were excluded, as were those with pneumonia or other respiratory tract infections not resolved ≤14 days or ≤7 days, respectively, prior to screening.

### Study endpoints

Presence of CMH at baseline (CMH+ status) was defined as patients reporting cough (St George's Respiratory Questionnaire [SGRQ] question 1) and sputum (SGRQ question 2) on most or several days per week (baseline score of 1 or 2 for each question).^10^, ^11^ This definition has been shown to be an independent predictor of severe exacerbation frequency unlike the classic definition of cough and phlegm for ≥3 months per year for ≥2 consecutive years.^12^ Subgroups based on CMH status (CMH +/−) were derived post hoc from the intent‐to‐treat (ITT) population and study endpoints were assessed by CMH status at baseline. Only patients with non‐missing data on SGRQ question 1 and 2 at baseline were included in this analysis.

Endpoints assessed by baseline CMH status in this analysis included rate of on‐treatment moderate/severe and severe exacerbations; change from baseline in trough FEV_1_, SGRQ total score and CAT score at Week 52 and over time; proportion of SGRQ responders (patients with a decrease in SGRQ total score from baseline of ≥4 points) at Week 52; proportion of CAT responders (patients with a decrease in CAT total score from baseline of ≥2 points) at Week 52; and all‐cause mortality up to Week 52. Safety endpoints included incidence of adverse events of special interest (AESIs), including cardiovascular effects, local corticosteroid effects and pneumonia.

Moderate exacerbations were defined as requiring treatment with oral/systemic corticosteroids and/or antibiotics. Severe exacerbations were defined as requiring hospitalization or resulting in death.

### Statistical analysis

Study population characteristics and efficacy and safety endpoints were assessed in the ITT population. On‐treatment moderate/severe and severe exacerbation rates by baseline CMH status and by treatment as an interaction were analyzed using a generalized linear model assuming a negative binomial distribution and covariates of treatment group, sex, exacerbation history (≤1, ≥2; moderate/severe), smoking status (at screening), geographical region, baseline SGRQ CMH group, post‐bronchodilator percent predicted FEV_1_ (at screening) and treatment group by baseline SGRQ CMH subgroup interaction. Change from baseline in trough FEV_1_, SGRQ total score and CAT score by baseline CMH status were analyzed using a repeated measures model. The proportions of SGRQ and CAT responders at Week 52 by baseline CMH status were analyzed using a generalized linear mixed model with a logit link function. Statistical models included multiple covariates, including smoking status at screening, with further details listed in Appendix S1 in the Supporting Information. Safety endpoints were summarized descriptively.

## RESULTS

### Study population

The ITT population included 10,355 patients; of which 10,250 reported baseline SGRQ data and were included in this analysis. Of those, 6383 (62%) were CMH+ (Table 1). History of exacerbations, body mass index and lung function were similar across subgroups; however, there was a greater proportion of current smokers in the CMH+ versus the CMH− subgroup (43% vs. 21%), and higher baseline CAT scores (19.8 vs. 15.5). Baseline SGRQ total score was also higher in the CMH+ subgroup compared with the CMH− subgroup (53.6 vs. 45.8) with this direction of difference seen across all SGRQ domains (Table S1 in the Supporting Information).

**TABLE 1:** Baseline characteristics by baseline CMH status

	CMH+				CMH−			
Characteristic	FF/UMEC/VI (*N* = 2539)	FF/VI (*N* = 2580)	UMEC/VI (*N* = 1264)	Total (*N* = 6383)	FF/UMEC/VI (*N* = 1569)	FF/VI (*N* = 1512)	UMEC/VI (*N* = 786)	Total (*N* = 3867)
Age, mean (SD) years	64.9 (8.2)	64.7 (8.3)	64.7 (8.3)	64.8 (8.3)	66.1 (8.3)	66.3 (8.1)	66.1 (8.2)	66.2 (8.2)
Male sex, *n* (%)	1737 (68)	1765 (68)	838 (66)	4340 (68)	1000 (64)	961 (64)	503 (64)	2464 (64)
BMI^a^, mean (SD) kg/m^2^	26.6 (6.3)	26.5 (6.0)	26.4 (5.8)	26.5 (6.1)	26.7 (6.1)	26.9 (6.2)	26.9 (5.9)	26.8 (6.1)
Post‐bronchodilator FEV_1_ ^b^, mean (SD) ml	1291 (505)	1284 (495)	1275 (490)	1285 (498)	1251 (495)	1254 (493)	1271 (494)	1256 (494)
Post‐bronchodilator FEV_1_ ^c^ % predicted, *n* (%)								
<30%	411 (16)	399 (15)	203 (16)	1013 (16)	250 (16)	237 (16)	112 (14)	599 (15)
30–<40%	584 (23)	625 (24)	311 (25)	1520 (24)	344 (22)	329 (22)	176 (22)	849 (22)
40–<50%	594 (23)	675 (26)	322 (25)	1591 (25)	391 (25)	384 (25)	197 (25)	972 (25)
≥50%	948 (37)	880 (34)	428 (34)	2256 (35)	581 (37)	562 (37)	300 (38)	1443 (37)
Exacerbations in the previous year, *n* (%)								
≥2 moderate or ≥1 severe	1781 (70)	1789 (69)	889 (70)	4459 (70)	1140 (73)	1073 (71)	552 (70)	2765 (72)
≥2 moderate/severe	1399 (55)	1364 (53)	692 (55)	3455 (54)	871 (56)	834 (55)	438 (56)	2143 (55)
Current smokers, *n* (%)	1079 (42)	1087 (42)	550 (44)	2716 (43)	345 (22)	314 (21)	172 (22)	831 (21)
SGRQ total score at baseline, mean (SD)	54.0 (16.3)	53.4 (16.6)	53.1 (16.0)	53.6 (16.3)	45.6 (16.3)	46.1 (16.8)	45.6 (16.8)	45.8 (16.6)
CAT score^d^, mean (SD)	19.9 (6.8)	19.9 (6.8)	19.7 (6.6)	19.8 (6.8)	15.4 (6.4)	15.7 (6.5)	15.5 (6.5)	15.5 (6.4)
COPD medication at screening^e^, *n* (%)								
ICS + LAMA + LABA	1026 (40)	1043 (40)	554 (44)	2623 (41)	622 (40)	586 (39)	298 (38)	1506 (39)
ICS + LABA	797 (31)	813 (32)	382 (30)	1992 (31)	546 (35)	513 (34)	262 (33)	1321 (34)
LAMA + LABA	229 (9)	213 (8)	117 (9)	559 (9)	159 (10)	133 (9)	76 (10)	368 (10)
LAMA	202 (8)	245 (9)	91 (7)	538 (8)	98 (6)	118 (8)	71 (9)	287 (7)
Blood eosinophil count^f^, mean (SD) 10^9^/L	0.221 (0.249)	0.228 (0.237)	0.227 (0.225)	0.225 (0.239)	0.217 (0.204)	0.216 (0.244)	0.226 (0.229)	0.219 (0.225)

Abbreviations: BMI, body mass index; CAT, COPD Assessment Test; CMH, chronic mucus hypersecretion; COPD, chronic obstructive pulmonary disease; FEV_1_, forced expiratory volume in 1 second; FF, fluticasone furoate; ICS, inhaled corticosteroid; LABA, long‐acting β_2_‐agonist; LAMA, long‐acting muscarinic antagonist; SD, standard deviation; SGRQ, St George's Respiratory Questionnaire; UMEC, umeclidinium and VI, vilanterol.

^a^ CMH+, total *n* = 6382, FF/UMEC/VI *n* = 2538; CMH−, total *n* = 3865, FF/UMEC/VI *n* = 1567.

^b^ CMH+, total *n* = 6319, FF/UMEC/VI, *n* = 2515, FF/VI *n* = 2549, UMEC/VI *n* = 1255; CMH−, total *n* = 3828, FF/UMEC/VI *n* = 1550, FF/VI *n* = 1500, UMEC/VI *n* = 778.

^c^ CMH+, total *n* = 6380, FF/UMEC/VI, *n* = 2537, FF/VI *n* = 2579, UMEC/VI *n* = 1264; CMH−, total *n* = 3863, FF/UMEC/VI *n* = 1566, FF/VI *n* = 1512, UMEC/VI *n* = 785.

^d^ CMH+, total *n* = 6327, FF/UMEC/VI *n* = 2523, FF/VI *n* = 2551, UMEC/VI *n* = 1253; CMH−, total *n* = 3829, FF/UMEC/VI *n* = 1553, FF/VI *n* = 1495, UMEC/VI *n* = 781.

^e^ Between date of screening −3 days and screening (inclusive).

^f^ CMH+, total *n* = 6368, FF/UMEC/VI *n* = 2532, FF/VI *n* = 2574, UMEC/VI *n* = 1262; CMH−, total *n* = 3860, FF/UMEC/VI *n* = 1568, FF/VI *n* = 1509, UMEC/VI *n* = 783.

### On‐treatment exacerbation rates

**FIGURE 1:**
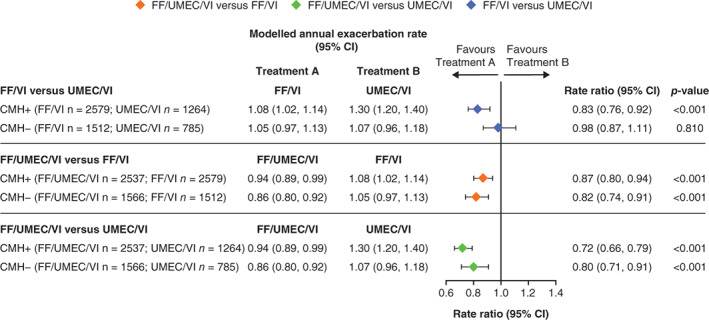
On‐treatment moderate/severe exacerbation rates by baseline CMH status (CMH+, CMH−). CI, confidence interval; CMH, chronic mucus hypersecretion; FF, fluticasone furoate; UMEC, umeclidinium and VI, vilanterol

On‐treatment moderate/severe exacerbation rates were numerically higher in the CMH+ subgroup than the CMH− subgroup for all treatment groups (Figure 1). FF/UMEC/VI significantly reduced the rate of on‐treatment moderate/severe exacerbations compared with FF/VI and UMEC/VI in both CMH subgroups (Figure 1), while FF/VI demonstrated a significant reduction versus UMEC/VI in the CMH+ subgroup (rate ratio: 0.83; 95% confidence interval [CI]: 0.76, 0.92; *p* < 0.001), but not the CMH− subgroup (Figure 1). There was no evidence of a statistically significant interaction between CMH subgroup and treatment effect (*p* = 0.112).

**FIGURE 2:**
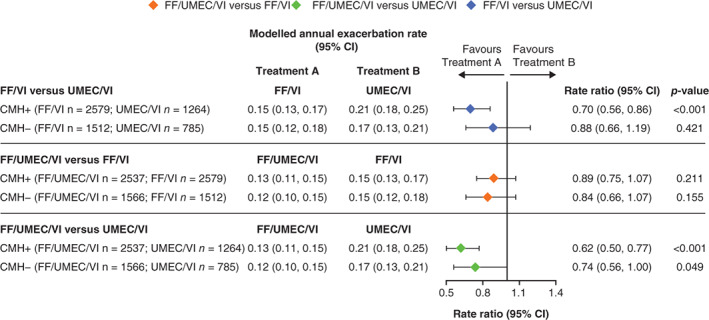
On‐treatment severe exacerbation rates by baseline CMH status (CMH+, CMH−). CI, confidence interval; CMH, chronic mucus hypersecretion; FF, fluticasone furoate; UMEC, umeclidinium and VI, vilanterol

FF/UMEC/VI also significantly reduced severe exacerbation rates versus UMEC/VI in both CMH subgroups (Figure 2). Rate ratios for severe exacerbations favoured FF/UMEC/VI over FF/VI in both CMH subgroups but were not statistically significant (Figure 2). FF/VI significantly reduced the rate of on‐treatment severe exacerbations compared with UMEC/VI in the CMH+ subgroup (rate ratio: 0.70; 95% CI: 0.56, 0.86; *p* < 0.001) but not in the CMH− subgroup (Figure 2).

### Lung function

**FIGURE 3:**
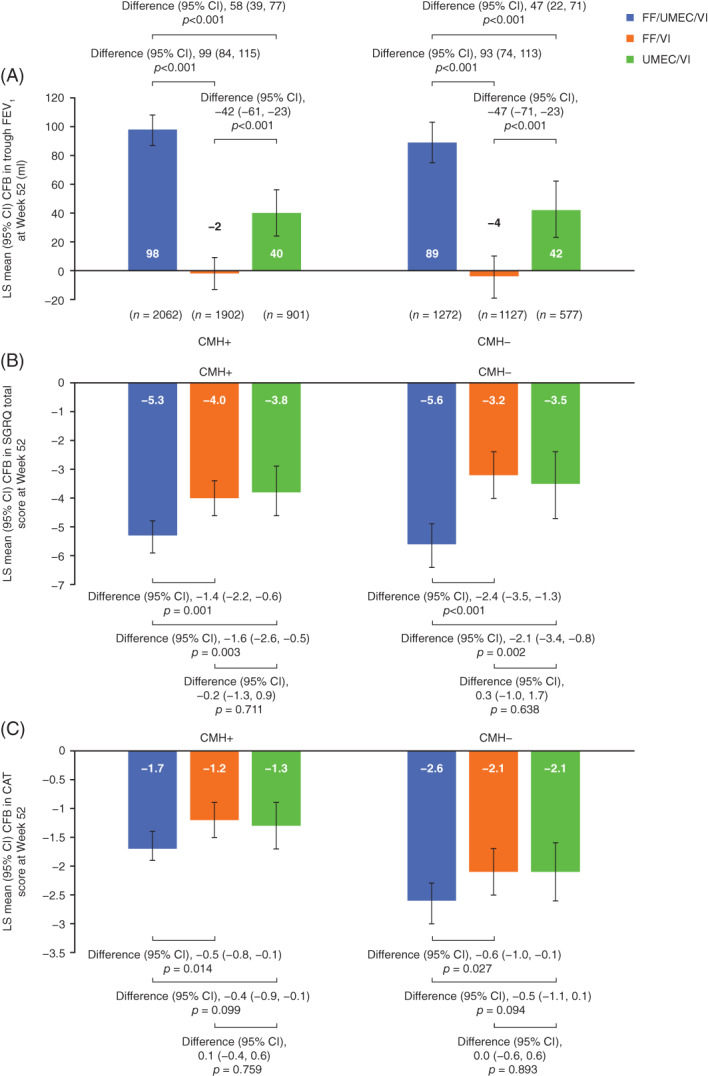
Change from baseline in (A) trough FEV_1_, (B) in SGRQ total score and (C) CAT score at Week 52 by baseline CMH status (CMH+, CMH−). Analysis performed using a repeated measures model with covariates of treatment group, smoking status (screening), geographical region, baseline SGRQ CMH status, visit, baseline, baseline by visit, treatment group by visit, treatment group by baseline SGRQ CMH status and treatment group by visit by baseline SGRQ CMH status interactions. CAT, COPD Assessment Test; CFB, change from baseline; CI, confidence interval; CMH, chronic mucus hypersecretion; FEV_1_, forced expiratory volume in 1 s; FF, fluticasone furoate; LS, least squares; SGRQ, St George's respiratory questionnaire; UMEC, umeclidinium and VI, vilanterol

Lung function improvements from baseline at Week 52 were significantly greater in patients receiving FF/UMEC/VI compared with both dual therapies as well as in patients receiving UMEC/VI versus FF/VI, regardless of CMH status at baseline (Figure 3A). This was consistently seen at all time points (Figure S1 in the Supporting Information).

### Health status

At Week 52, significant improvements from baseline in SGRQ total score were observed with FF/UMEC/VI compared with both dual therapies across both CMH subgroups (Figure 3B); this was consistently observed at all time points (Figure S2 in the Supporting Information). Treatment with FF/UMEC/VI also resulted in a significantly greater proportion of SGRQ responders compared with either dual therapy, regardless of CMH status (Table 2).

**TABLE 2:** Proportion of SGRQ^a^ and CAT^b^ responders at week 52 by baseline CMH status

	CMH+			CMH−		
	FF/UMEC/VI (*N* = 2539)	FF/VI (*N* = 2580)	UMEC/VI (*N* = 1264)	FF/UMEC/VI (*N* = 1569)	FF/VI (*N* = 1512)	UMEC/VI (*N* = 786)
SGRQ responders, *n* (%)	1088 (43)	899 (35)	457 (36)	635 (40)	491 (32)	239 (30)
FF/UMEC/VI versus comparator, OR (95% CI); *p*‐value		1.40 (1.25, 1.57); <0.001	1.32 (1.14, 1.51); <0.001		1.44 (1.24, 1.67); <0.001	1.58 (1.31, 1.89); <0.001
FF/VI versus UMEC/VI, OR (95% CI); *p*‐value			0.94 (0.82, 1.09); 0.394			1.10 (0.91, 1.32); 0.338
CAT^c^, responders, *n* (%)	1071 (42)	928 (36)	451 (36)	627 (40)	536 (38)	279 (36)
FF/UMEC/VI versus comparator, OR (95% CI); *p*‐value		1.30 (1.16, 1.46); <0.001	1.31 (1.14, 1.51); <0.001		1.15 (0.99, 1.33); 0.068	1.23 (1.03, 1.48); 0.023
FF/VI versus UMEC/VI, OR (95% CI); *p*‐value			1.01 (0.87, 1.16); 0.928			1.08 (0.89, 1.28); 0.439

Abbreviations: CAT, COPD Assessment Test; CI, confidence interval; CMH, chronic mucus hypersecretion; FF, fluticasone furoate; OR, odds ratio; SGRQ, St George's Respiratory Questionnaire; UMEC, umeclidinium; VI, vilanterol.

^a^ Response was defined as an SGRQ total score decrease of ≥4 points from baseline. Patients were not included in the SGRQ responder analysis if their SGRQ total score was missing at the Week 52 visit.

^b^ Response was defined as a CAT total score decrease of ≥2 points from baseline. Patients were not included in the CAT responder analysis if baseline CAT score was missing.

^c^ CMH+, FF/UMEC/VI *n* = 2523, FF/VI *n* = 2551, UMEC/VI *n* = 1253; CMH−, FF/UMEC/VI *n* = 1553, FF/VI *n* = 1495, UMEC/VI *n* = 781.

At Week 52, and consistently at all time points, significant improvements in CAT score were observed with FF/UMEC/VI compared with FF/VI across both CMH subgroups (Figure 3C and Figure S3 in the Supporting Information). FF/UMEC/VI significantly improved CAT score versus UMEC/VI at Week 4 in both CMH subgroups but the between‐treatment difference was no longer significant at Week 28 or 52 (Figure S3 in the Supporting Information). Treatment with FF/UMEC/VI resulted in a significantly greater proportion of CAT responders at Week 52 compared with UMEC/VI in both CMH subgroups, and compared with FF/VI in the CMH+ subgroup (Table 2).

Both FF/VI and UMEC/VI improved SGRQ total score and CAT score from baseline at Week 52 irrespective of CMH status, with no significant difference between treatments; however, CAT score improvements from baseline were numerically lower in the CMH+ versus the CMH− subgroup (Figure 3B,C). At Week 52, there was no difference in the proportion of SGRQ and CAT responders with FF/VI and UMEC/VI in either CMH subgroup (Table 2).

### All‐cause mortality

All‐cause mortality incidence was numerically higher in the CMH+ subgroup than the CMH− subgroup in all treatment arms (1.3%–2.1% and 1.1%–1.4%, respectively; Table 3). In the CMH+ subgroup, the incidence of all‐cause mortality was numerically higher in the UMEC/VI treatment arm compared with both inhaled corticosteroid (ICS)‐containing arms (Table 3).

**TABLE 3:** Safety profile by baseline CMH status

	CMH+						CMH−					
	FF/UMEC/VI (*N* = 2539)		FF/VI (*N* = 2580)		UMEC/VI (*N* = 1264)		FF/UMEC/VI (*N* = 1569)		FF/VI (*N* = 1512)		UMEC/VI (*N* = 786)	
AESI (any event)	*n* (%)	Rate [#]	*n* (%)	Rate [#]	*n* (%)	Rate [#]	*n* (%)	Rate [#]	*n* (%)	Rate [#]	*n* (%)	Rate [#]
Anticholinergic syndrome (SMQ)	120 (5)	62.8 [143]	93 (4)	48.7 [105]	44 (3)	51.3 [53]	60 (4)	55.7 [78]	44 (3)	43.4 [55]	26 (3)	43.2 [28]
Asthma/bronchospasm (SMQ)	19 (<1)	8.3 [19]	20 (<1)	9.7 [21]	5 (<1)	4.8 [5]	8 (<1)	6.4 [9]	14 (<1)	11.0 [14]	11 (1)	17.0 [11]
Cardiovascular effects	291 (11)	184.8 [421]	286 (11)	166.5 [359]	149 (12)	187.6 [194]	155 (10)	140.0 [196]	141 (9)	142.0 [180]	74 (9)	135.7 [88]
Decreased BMD	63 (2)	36.4 [83]	46 (2)	23.2 [50]	23 (2)	25.1 [26]	34 (2)	26.4 [37]	39 (3)	33.9 [43]	13 (2)	23.1 [15]
Hyperglycaemia/new onset diabetes mellitus (SMQ)	90 (4)	47.8 [109]	67 (3)	34.8 [75]	48 (4)	51.3 [53]	60 (4)	46.4 [65]	50 (3)	44.2 [56]	24 (3)	38.6 [25]
Hypersensitivity	107 (4)	54.9 [125]	115 (4)	60.3 [130]	54 (4)	56.1 [58]	89 (6)	73.6 [103]	80 (5)	74.1 [94]	41 (5)	66.3 [43]
LRTI excluding pneumonia	126 (5)	64.5 [147]	118 (5)	68.6 [148]	71 (6)	81.2 [84]	73 (5)	60.7 [85]	79 (5)	71.8 [91]	36 (5)	67.9 [44]
Local corticosteroid effects	210 (8)	117.2 [267]	187 (7)	106.6 [230]	64 (5)	79.3 [82]	124 (8)	110.7 [155]	112 (7)	108.9 [138]	44 (6)	83.3 [54]
Ocular effects	33 (1)	15.8 [36]	20 (<1)	9.7 [21]	11 (<1)	10.6 [11]	21 (1)	17.9 [25]	25 (2)	23.7 [30]	14 (2)	21.6 [14]
Pneumonia	204 (8)	100.5 [229]	186 (7)	95.5 [206]	60 (5)	61.9 [64]	111 (7)	89.3 [125]	104 (7)	98.6 [125]	37 (5)	61.7 [40]
All‐cause mortality, *n* (%)^a^	32 (1.3)	N/A	33 (1.3)	N/A	26 (2.1)	N/A	18 (1.2)	N/A	16 (1.1)	N/A	11 (1.4)	N/A

*Note*: *n* = Number of patients, # = Number of events. Rate is event rate per 1000 patient‐years, calculated as the number of events × 1000, divided by the total duration at risk.

Abbreviations: AESI, adverse event of special interest; BMD, bone mineral density; CMH, chronic mucus hypersecretion; FF, fluticasone furoate; LRTI, lower respiratory tract infection; N/A, not available; SMQ, Standardized MedDRA Query; UMEC, umeclidinium; VI, vilanterol.

a On‐treatment deaths occurring between study treatment start date and 7 days after study treatment stop date (inclusive).

### Safety

The AESI profile of FF/UMEC/VI was similar to that of the dual therapies in both subgroups (Table 3). Pneumonia was reported in a small proportion of patients, with similar incidence in CMH+ and CMH− subgroups (Table 3). Pneumonia incidence was numerically higher in ICS‐containing treatment arms than in the UMEC/VI arm, irrespective of CMH status at baseline. The incidence of cardiovascular effects was numerically higher in the CMH+ subgroup compared with the CMH− subgroup in all treatment arms (Table 3; Table S2 in the Supporting Information), and the incidence of hypersensitivity events was slightly lower in the CMH+ subgroup compared with the CMH− subgroup in all treatment arms (Table 3; Table S2 in the Supporting Information).

## DISCUSSION

In this post hoc analysis, FF/UMEC/VI reduced on‐treatment exacerbation rates and improved lung function and health status versus FF/VI and UMEC/VI in patients with symptomatic COPD and a history of exacerbations irrespective of their CMH status at baseline.

At baseline both subgroups were similar regarding exacerbation history and lung function. However, patients with CMH at baseline were more likely to be current smokers and have worse health status compared with those without CMH. On‐treatment moderate/severe exacerbation rates were significantly lower for patients receiving FF/UMEC/VI versus FF/VI and UMEC/VI, regardless of CMH status. Additionally, when comparing dual therapies, FF/VI reduced exacerbation rates versus UMEC/VI, albeit only in the CMH+ subgroup. These results suggest that ICS is likely to be driving this benefit, despite the potential blunting effect of smoking on the clinical response to ICS in COPD.^13^, ^14^ This is in line with a recent post hoc analysis of IMPACT, which found FF/UMEC/VI improved clinical outcomes versus dual therapy in patients with symptomatic COPD and a history of exacerbations, regardless of smoking status.^15^

While both subgroups had similar exacerbation history, patients in the CMH+ subgroup had numerically higher rates of on‐treatment moderate/severe and severe exacerbations during the study compared with the CMH− subgroup, with the greatest difference between CMH subgroup seen in the UMEC/VI treatment arm. This observation provides further support that CMH as defined using the SGRQ respiratory items on chronic cough and phlegm may be a predictor for the risk of future exacerbations. Other studies that have shown an association between CMH defined using the SGRQ criteria utilized in the current analysis and an increased risk of exacerbation, hospitalization and death.^4^, ^16^, ^17^, ^18^, ^19^ This finding may be explained by CMH+ patients having a greater number of resident bacteria^7^ and pre‐existing low‐grade bronchial reactivity compared with CMH− patients.^7^, ^20^ In addition, their airway obstruction may be more proximal and vulnerable to environmental stimuli such as particulate air pollution.^21^, ^22^ It is worth noting that in IMPACT, baseline exacerbation history was determined retrospectively by physician‐confirmed exacerbations, rather than patient self‐reported events. On‐treatment exacerbations, however, were judged by the investigators, based on patients' symptoms reported via an eDiary. As such, not all exacerbations during the 12 months prior to study entry may have been recorded.

As shown in this analysis, treatment with triple therapy resulted in improvements in outcomes irrespective of CMH baseline status compared with either dual therapy. A pooled analysis of the oral selective phosphodiesterase‐4 inhibitor roflumilast has shown certain COPD phenotypes such as chronic bronchitis with/without concurrent ICS may be more likely to respond to therapy.^23^ Together, this suggests that CMH status could be used as an enrichment biomarker in future clinical trials for dual therapies to assess whether this may be a treatment biomarker similar to blood eosinophils.^24^, ^25^, ^26^

As would be expected from the definition of CMH used in this study (i.e., using SGRQ respiratory items on chronic cough and phlegm), on‐treatment CAT and SGRQ scores in the CMH+ group suggested worse health‐related quality of life throughout the study. This is in line with previous studies, that have found that CMH can result in worse quality of life in patients with COPD.^16^, ^17^ Additionally, the PLATINO study found that patients with COPD and chronic bronchitis had worse lung function and health status, and more exacerbations than patients without chronic bronchitis.^27^

For all‐cause mortality, incidence was numerically higher in the CMH+ subgroup than the CMH− subgroup in all treatment arms, likely due to the worse health status and numerically higher on‐treatment moderate/severe exacerbation rates seen in the CMH+ subgroup. In the CMH+ subgroup, the incidence of all‐cause mortality was numerically higher in the UMEC/VI treatment arm compared with both ICS‐containing arms, which is consistent with the mortality benefit seen in the IMPACT trial among patients treated with FF/UMEC/VI versus UMEC/VI.^8^ However, given the low numbers of events in the overall trial, subgroup analyses on mortality should be interpreted with caution. The safety profile of triple therapy was similar to dual therapy in both subgroups. Occurrence of pneumonia was low across all treatment groups, although a numerically higher incidence was seen with FF‐containing therapies versus UMEC/VI in both CMH subgroups. The incidence of cardiovascular AESIs was numerically higher, and that of hypersensitivity AESIs numerically lower, in the CMH+ versus the CMH− subgroup in all treatment arms.

The Medical Research Council (MRC) defines CMH as symptoms of chronic cough and sputum for ≥3 months over at least a year for 2 consecutive years^28^; however, the current analysis defined CMH using SGRQ respiratory items on chronic cough and phlegm (SGRQ question 1 or 2). Despite using a different definition this approach has previously been validated in an analysis of the COPDGene cohort, which showed that the use of the SGRQ identified CMH with a high sensitivity and specificity compared with the classical MRC definition and was a similar, if not better, predictor of future severe exacerbations.^10^, ^12^ Another study has shown that the CAT questionnaire could also be used to assess CMH status.^29^ CAT is short and simple, and it is routinely carried out in clinical practice to evaluate and monitor health status in patients with COPD, making it a potential valuable tool to assess CMH status and thus personalize COPD treatment in clinical practice.

This analysis of the IMPACT trial is not without its limitations. Analyses were exploratory and conducted post hoc and the IMPACT trial was not powered to analyse endpoints by CMH status. Additionally, patients were selected based on history of exacerbation, and thus findings from this analysis may not apply to a population at low risk of exacerbations. Strengths of the study include the large sample size of the IMPACT trial, which allowed for this subgroup analysis and provided the opportunity to gain insight into whether there is another phenotype besides blood eosinophil levels that may predict the effect of FF/UMEC/VI in COPD.

In conclusion, FF/UMEC/VI reduced moderate/severe exacerbation rates and improved lung function and health status compared with FF/VI and UMEC/VI in patients with symptomatic COPD and a history of exacerbations, regardless of their CMH status at baseline, with a similar safety profile. These results highlight the favourable benefit: risk profile of once‐daily FF/UMEC/VI across a multitude of COPD endpoints, regardless of CMH status at baseline. Differences in clinical efficacy and safety outcomes between CMH+ and CMH− subgroups were observed when comparing the two dual therapies. Future studies should seek to determine whether treatment alleviates the defining symptoms on CMH over time. The analysis presented here highlights that phenotyping patients may be useful in future clinical trials and that CMH status could be used to help optimize pharmacological management approaches for this population.

## AUTHOR CONTRIBUTION

**Philip J. Thompson:** Visualization (equal); writing – original draft (equal); writing – review and editing (equal). **Gerard Criner:** Investigation (lead); visualization (equal); writing – original draft (equal); writing – review and editing (equal). **Mark T. Dransfield:** Visualization (equal); writing – original draft (equal); writing – review and editing (equal). **David M. G. Halpin:** Investigation (lead); visualization (equal); writing – original draft (equal); writing – review and editing (equal). **MeiLan K. Han:** Investigation (lead); visualization (equal); writing – original draft (equal); writing – review and editing (equal). **David A. Lipson:** Conceptualization (lead); data curation (lead); investigation (lead); methodology (lead); visualization (equal); writing – original draft (equal); writing – review and editing (equal). **Ghassan J. Maghzal:** Visualization (equal); writing – original draft (equal); writing – review and editing (equal). **Fernando Martinez:** Investigation (equal); visualization (equal); writing – original draft (equal); writing – review and editing (equal). **Dawn Midwinter:** Formal analysis (equal); visualization (equal); writing – original draft (equal); writing – review and editing (equal). **Dave Singh:** Investigation (equal); visualization (equal); writing – original draft (equal); writing – review and editing (equal). **Lee Tombs:** Formal analysis (equal); visualization (equal); writing – original draft (equal); writing – review and editing (equal). **Robert A. Wise:** Investigation (equal); visualization (equal); writing – original draft (equal); writing – review and editing (equal).

## CONFLICTS OF INTEREST

This study was funded by GSK (study number CTT116855). The funders of the study had a role in the study design, data analysis, data interpretation and writing of the report. Philip J. Thompson received consultancy fees from AstraZeneca, Boehringer Ingelheim, CSL Behring, Grifols, GSK and Novartis. Gerard J. Criner received personal fees from GSK, Boehringer Ingelheim, Novartis, Almirall, AstraZeneca, Chiesi, Pulmonx, Olympus, Nuvaira, Eolo, CSA Medical, HGE Technologies and Verona. Mark T. Dransfield received consulting fees from Pumonx and Teva, and personal fees and contracted clinical trial support from Boehringer Ingelheim, GSK, AstraZeneca and PneumRx/BTG. He received personal fees from Quark Pharmaceuticals as well as grant support from the American Lung Association, Department of Defence, Department of Veterans Affairs and NIH. Additionally, he received contracted clinical trial support from Novartis, Yungjin, Pulmonx, Boston Scientific, Gala and Nuvaira. David M. G. Halpin received personal fees from AstraZeneca, Boehringer Ingelheim, Chiesi, GSK, Novartis and Pfizer. MeiLan K. Han reports personal fees from GSK, AstraZeneca, Boehringer Ingelheim, Cipla, Chiesi, Novartis, Pulmonx, Teva, Verona, Merck, Mylan, Sanofi, DevPro, Aerogen, Polarian, Regeneron, United Therapeutics, Altesa Biopharma, UpToDate, Medscape and Integrity. She has received either in‐kind research support or funds paid to the institution from the NIH, Novartis, Sunovion, Nuvaira, Sanofi, AstraZeneca, Boehringer Ingelheim, Gala Therapeutics, Biodesix, the COPD Foundation and the American Lung Association. She has participated in Data Safety Monitoring Boards for Novartis and Medtronic with funds paid to the institution. She has received stock options from Meissa Vaccines and Altesa Biopharma. David A. Lipson, Ghassan J. Maghzal and Dawn Midwinter are employees and shareholders at GSK. Fernando J. Martinez received consulting fees from AstraZeneca, Boehringer Ingelheim, Chiesi, CSL Behring, Gala, GSK, Novartis, Polarean, Pulmonx, Sanofi/Regeneron, Sunovion, Teva, Theravance/Viatris and Verona; grant support from AstraZeneca, Chiesi, GSK and Sanofi/Regeneron; honoraria from UpToDate for participation in COPD CME activities; and participated in an event adjudication committee for MedTronic. Dave Singh received consulting fees from Aerogen, AstraZeneca, Boehringer Ingelheim, Chiesi, Cipla, CSL Behring, Epiendo, Genentech, GSK, Glenmark, Gossamerbio, Kinaset, Menarini, Novartis, Pulmatrix, Sanofi, Synairgen, Teva, Therevance and Verona. Lee Tombs is a contingent worker on assignment at GSK. Robert A. Wise received consulting fees for participation on Data Safety Monitoring Boards or Advisory Boards from AstraZeneca/MedImmune, Boehringer Ingelheim, Contrafect, Roche‐Genetech, Bristol Myers Squibb, Merck, Verona, Theravance, AbbVie, GSK, Chemerx, Kiniksa, Savara, Galderma, Kamada, Pulmonx, Kinevant, Vaxart, Polarean, Chiesi, 4D Pharma and Puretech; and received grant support from AstraZeneca, Boehringer Ingelheim, Genentech, Verona, 4DX imaging and Sanofi. He has received payment for expert testimony from the United States Government and Genentech; and support for attending meetings and/or travel from AstraZeneca. Additionally, he has received editorial support from GSK, AstraZeneca, Boehringer Ingelheim and Merck Foundation; and has served on the Board of Directors/Medical and Scientific Advisory Committee for the COPD Foundation, and on a Scientific Advisory Board for the American Lung Association.

## HUMAN ETHICS APPROVAL DECLARATION

The study was conducted in accordance with Good Clinical Practice guidelines and the provisions of the Declaration of Helsinki. It received approval from the institutional review boards and/or human research ethics committees of all investigator sites for the IMPACT study. All patients provided written informed consent.

Clinical trial registration: NCT02164513 at https://clinicaltrials.gov

## Supporting information


Appendix S1

**Figure S1** Change from baseline in trough FEV_1_ by baseline CMH status.

**Figure S2** Change from baseline in SGRQ total score by baseline CMH status.

**Figure S3** Change from baseline in CAT score by baseline CMH status.

**Table S1** Summary of baseline SGRQ scores by CMH status.

**Table S2** Incidence of on‐treatment AESIs.

## Data Availability

Anonymized individual participant data and study documents can be requested for further research from www.clinicalstudydatarequest.com.
