# Supplementary material for: Effect of chronic mucus hypersecretion on treatment responses to inhaled therapies in patients with chronic obstructive pulmonary disease: Post hoc analysis of the IMPACT trial
Source: Respirology. 2022 Aug 15;27(12):1034–44. doi: 10.1111/resp.14339 (PMC9804213; doi:10.1111/resp.14339)
Supplement: Supplementary file 1 — Appendix S1 [file RESP-27-1034-s003.docx]

# Appendix S1

# Methods

## Statistical analyses

On-treatment moderate/severe and severe exacerbation rates by baseline CMH status and by treatment as an interaction were analysed using a generalized linear model assuming a negative binomial distribution with covariates of treatment group, sex, exacerbation history (≤1, ≥2 moderate/severe), smoking status at screening, geographical region, baseline SGRQ CMH subgroup, post-bronchodilator % predicted FEV_1_ at screening and treatment group by baseline SGRQ CMH subgroup interaction. Change from baseline in trough FEV_1_, SGRQ total score and CAT score by baseline CMH status were analysed using a repeated measures model with covariates of treatment group, smoking status at screening, geographical region, baseline SGRQ CMH status, visit, baseline parameter, baseline by visit, treatment group by visit, treatment group by baseline SGRQ CMH status and treatment group by visit by baseline SGRQ CMH status interactions. The proportions of SGRQ and CAT responders at Week 52 by baseline CMH status were analysed using a generalized linear mixed model with a logit link function with covariates of treatment group, smoking status at screening, geographical region, baseline SGRQ CMH status, visit, baseline, baseline by visit, treatment group by visit, treatment group by baseline SGRQ CMH status and treatment group by visit by baseline SGRQ CMH status interactions.
