# Supplementary material for: Effect of chronic mucus hypersecretion on treatment responses to inhaled therapies in patients with chronic obstructive pulmonary disease: Post hoc analysis of the IMPACT trial
Source: Respirology. 2022 Aug 15;27(12):1034–44. doi: 10.1111/resp.14339 (PMC9804213; doi:10.1111/resp.14339)
Supplement: Supplementary file 4 — Figure S3 Change from baseline in CAT score by baseline CMH status. [file RESP-27-1034-s001.docx]

**Figure S3.** Change from baseline in CAT score by baseline CMH status

**
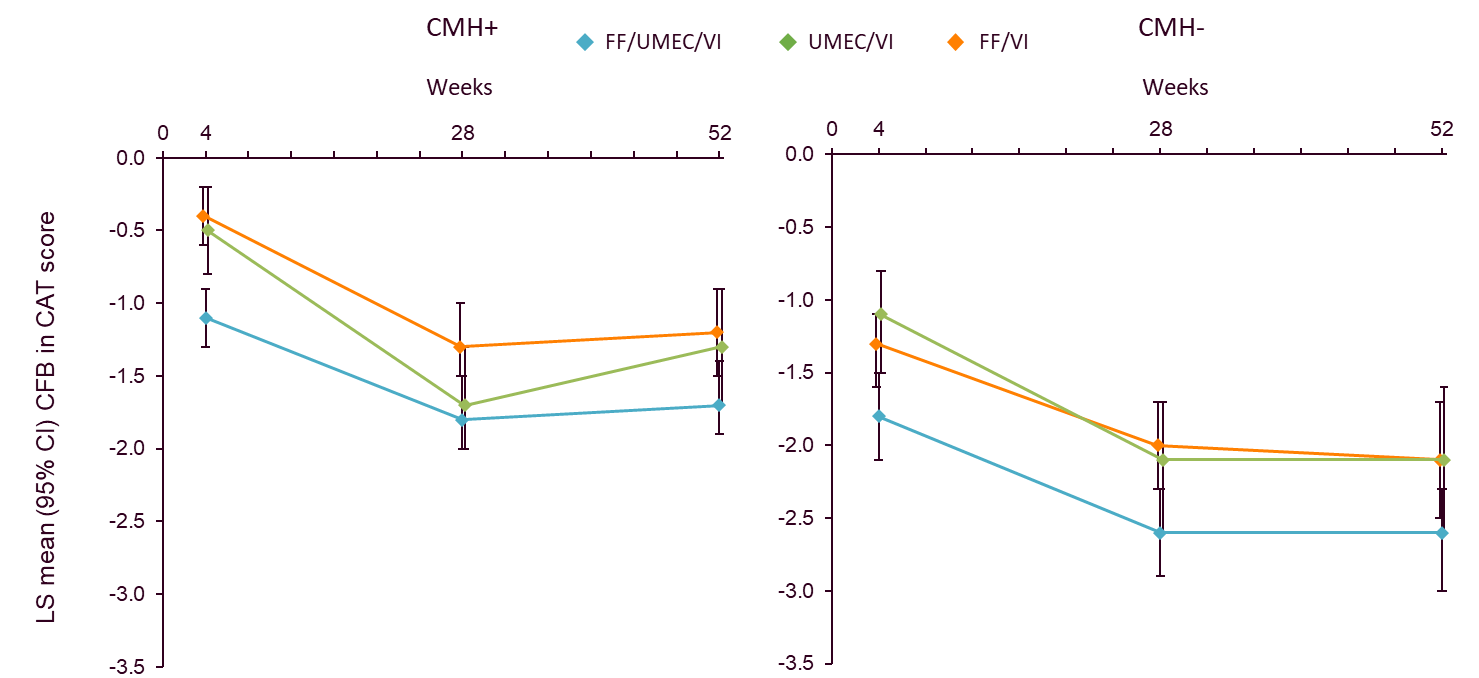
**

CFB, change from baseline; CAT, COPD Assessment Test; CFB, change from baseline; CI, confidence interval; CMH, chronic mucus hypersecretion; FF, fluticasone furoate; LS, least squares; UMEC, umeclidinium; VI, vilanterol.
