# Supplementary material for: Effect of chronic mucus hypersecretion on treatment responses to inhaled therapies in patients with chronic obstructive pulmonary disease: Post hoc analysis of the IMPACT trial
Source: Respirology. 2022 Aug 15;27(12):1034–44. doi: 10.1111/resp.14339 (PMC9804213; doi:10.1111/resp.14339)
Supplement: Supplementary file 5 — Table S1 Summary of baseline SGRQ scores by CMH status. [file RESP-27-1034-s005.docx]

**Table S1.** Summary of baseline SGRQ scores by CMH status

|  | **CMH+** | | | **CMH-** | | | |
| --- | --- | --- | --- | --- | --- | --- | --- |
|  | **FF/UMEC/VI**  **(N=2539)** | **FF/VI**  **(N=2580)** | **UMEC/VI**  **(N=1264)** | **FF/UMEC/VI**  **(N=1569)** | **FF/VI**  **(N=1512)** | **UMEC/VI**  **(N=786)** |  |
| **SGRQ Total score, mean (SD)** | 54.0 (16.30) | 53.4 (16.55) | 53.1 (15.97) | 45.6 (16.32) | 46.1 (16.82) | 45.6 (16.77) |  |
| **Symptoms domain, mean (SD)** | 73.7 (15.23) | 73.7 (15.17) | 73.2 (15.63) | 53.3 (16.27) | 53.7 (16.10) | 52.5 (16.28) |  |
| **Activity domain, mean (SD)** | 67.6 (18.85) | 66.5 (19.21) | 67.3 (18.47) | 63.2 (19.74) | 63.3 (20.25) | 63.2 (20.11) |  |
| **Impacts domain, mean (SD)** | 40.0 (19.33) | 39.6 (19.53) | 38.7 (18.72) | 32.9 (18.61) | 33.8 (18.93) | 33.3 (18.98) |  |

Note: Baseline is defined as the score recorded prior to dosing on Day 1.

CMH, chronic mucus hypersecretion; FF, fluticasone furoate; SGRQ, St George’s Respiratory Questionnaire; UMEC, umeclidinium; VI, vilanterol.
