# Supplementary material for: Effect of chronic mucus hypersecretion on treatment responses to inhaled therapies in patients with chronic obstructive pulmonary disease: Post hoc analysis of the IMPACT trial
Source: Respirology. 2022 Aug 15;27(12):1034–44. doi: 10.1111/resp.14339 (PMC9804213; doi:10.1111/resp.14339)
Supplement: Supplementary file 6 — Table S2 Incidence of on‐treatment AESIs. [file RESP-27-1034-s004.docx]

**Table S2.** Incidence of on-treatment AESIs

|  | **CMH+** | | | | | | **CMH-** | | | | | |
| --- | --- | --- | --- | --- | --- | --- | --- | --- | --- | --- | --- | --- |
|  | **FF/UMEC/VI**  **(N=2539)** | | **FF/VI**  **(N=2580)** | | **UMEC/VI**  **(N=1264)** | | **FF/UMEC/VI**  **(N=1569)** | | **FF/VI**  **(N=1512)** | | **UMEC/VI**  **(N=786)** | |
| **AESI (any event)^a^** | **n (%)** | **Rate [#]** | **n (%)** | **Rate [#]** | **n (%)** | **Rate [#]** | **n (%)** | **Rate [#]** | **n (%)** | **Rate [#]** | **n (%)** | **Rate [#]** |
| Any CV event | 291 (11) | 184.8 [421] | 286 (11) | 166.5 [359] | 149 (12) | 187.6 [194] | 155 (10) | 140.0 [196] | 141 (9) | 142.0 [180] | 74 (9) | 135.7 [88] |
| Cardiac arrhythmia | 108 (4) | 59.7 [136] | 112 (4) | 56.6 [122] | 50 (4) | 51.3 [53] | 43 (3) | 36.4 [51] | 48 (3) | 43.4 [55] | 30 (4) | 50.9 [33] |
| Cardiac failure (SMQ) | 88 (3) | 43.9 [100] | 87 (3) | 48.2 [104] | 47 (4) | 51.3 [53] | 49 (3) | 40.7 [57] | 39 (3) | 34.7 [ 44] | 21 (3) | 35.5 [23] |
| CNS haemorrhages and cerebrovascular conditions (SMQ) | 26 (1) | 12.7 [29] | 17 (<1) | 9.3 [20] | 8 (<1) | 7.7 [8] | 15 (<1) | 11.4 [16] | 10 (<1) | 8.7 [11] | 3 (<1) | 4.6 [3] |
| Hypertension (SMQ) | 67 (3) | 36.9 [84] | 73 (3) | 35.7 [77] | 36 (3) | 38.7 [40] | 45 (3) | 33.6 [47] | 40 (3) | 33.1 [42] | 18 (2) | 27.8 [18] |
| Ischemic heart disease (SMQ) | 59 (2) | 31.6 [72] | 33 (1) | 16.7 [36] | 36 (3) | 38.7 [40] | 21 (1) | 17.9 [25] | 24 (2) | 22.1 [28] | 11 (1) | 18.5 [12] |
| Any hypersensitivity event | 107 (4) | 54.9 [125] | 115 (4) | 60.3 [130] | 54 (4) | 56.1 [58] | 89 (6) | 73.6 [103] | 80 (5) | 74.1 [94] | 41 (5) | 66.3 [43] |
| Rhinitis allergic | 22 (<1) | 11.4 [26] | 29 (1) | 15.8 [34] | 16 (1) | 16.4 [17] | 13 (<1) | 12.1 [17] | 20 (1) | 16.6 [21] | 7 (<1) | 10.8 [7] |
| Rash | 23 (<1) | 11.0 [25] | 17 (<1) | 7.9 [17] | 13 (1) | 12.6 [13] | 20 (1) | 14.3 [20] | 17 (1) | 14.2 [18] | 3 (<1) | 4.6 [3] |
| Eczema | 11 (<1) | 5.3 [12] | 13 (<1) | 6.5 [14] | 4 (<1) | 4.8 [5] | 20 (1) | 14.3 [20] | 5 (<1) | 3.9 [5] | 9 (1) | 13.9 [9] |
| Any anticholinergic syndrome event (SMQ) | 120 (5) | 62.8 [ 143] | 93 (4) | 48.7 [105] | 44 (3) | 51.3 [53] | 60 (4) | 55.7 [78] | 44 (3) | 43.4 [55] | 26 (3) | 43.2 [28] |
| Dizziness | 34 (1) | 15.4 [35] | 25 (<1) | 11.6 [25] | 12 (<1) | 12.6 [13] | 19 (1) | 15.7 [22] | 10 (<1) | 7.9 [10] | 6 (<1) | 9.3 [6] |
| Pyrexia | 30 (1) | 14.9 [34] | 20 (<1) | 10.2 [22] | 13 (1) | 12.6 [13] | 19 (1) | 16.4 [23] | 16 (1) | 16.6 [21] | 8 (1) | 13.9 [9] |
| Any LRTI excluding pneumonia event | 126 (5) | 64.5 [147] | 118 (5) | 68.6 [148] | 71 (6) | 81.2 [84] | 73 (5) | 60.7 [85] | 79 (5) | 71.8 [91] | 36 (5) | 67.9 [44] |
| Bronchitis | 93 (4) | 47.0 [107] | 74 (3) | 46.4 [100] | 45 (4) | 50.3 [52] | 58 (4) | 47.1 [66] | 55 (4) | 48.9 [62] | 27 (3) | 50.9 [33] |
| LRTI | 22 (<1) | 11.0 [25] | 17 (<1) | 7.9 [17] | 13 (1) | 15.5 [16] | 7 (<1) | 5.7 [8] | 6 (<1) | 4.7 [6] | 4 (<1) | 7.7 [5] |
| Any local steroid effect event | 210 (8) | 117.2 [267] | 187 (7) | 106.6 [230] | 64 (5) | 79.3 [82] | 124 (8) | 110.7 [155] | 112 (7) | 108.9 [138] | 44 (6) | 83.3 [54] |
| Oral candidiasis | 96 (4) | 56.2 [128] | 103 (4) | 56.6 [122] | 26 (2) | 32.9 [34] | 64 (4) | 52.9 [74] | 42 (3) | 41.8 [53] | 15 (2) | 24.7 [16] |
| Oropharyngeal pain | 64 (3) | 29.4 [67] | 37 (1) | 21.3 [46] | 21 (2) | 23.2 [24] | 34 (2) | 28.6 [40] | 34 (2) | 28.4 [36] | 18 (2) | 37.0 [24] |
| Dysphonia | 21 (<1) | 9.2 [21] | 15 (<1) | 7.4 [16] | 10 (<1) | 10.6 [11] | 18 (1) | 13.6 [19] | 15 (<1) | 12.6 [16] | 4 (<1) | 6.2 [4] |
| Any ocular effect event | 33 (1) | 15.8 [36] | 20 (<1) | 9.7 [21] | 11 (<1) | 10.6 [11] | 21 (1) | 17.9 [25] | 25 (2) | 23.7 [30] | 14 (2) | 21.6 [14] |
| Glaucoma (SMQ) | 28 (1) | 12.7 [29] | 18 (<1) | 8.8 [19] | 9 (<1) | 8.7 [9] | 16 (1) | 13.6 [19] | 22 (1) | 21.3 [27] | 13 (2) | 20.1 [13] |
| Lens disorders (SMQ) | 24 (<1) | 11.9 [27] | 18 (<1) | 8.8 [19] | 8 (<1) | 7.7 [8] | 14 (<1) | 12.1 [17] | 21 (1) | 18.9 [24] | 11 (1) | 17.0 [11] |
| Any pneumonia event | 204 (8) | 100.5 [229] | 186 (7) | 95.5 [206] | 60 (5) | 61.9 [64] | 111 (7) | 89.3 [125] | 104 (7) | 98.6 [125] | 37 (5) | 61.7 [40] |
| Pneumonia | 191 (8) | 92.6 [211] | 173 (7) | 88.6 [191] | 57 (5) | 58.0 [60] | 106 (7) | 83.6 [117] | 90 (6) | 84.4 [107] | 36 (5) | 58.6 [38] |
| Any BMD decrease event | 63 (2) | 36.4 [83] | 46 (2) | 23.2 [50] | 23 (2) | 25.1 [26] | 34 (2) | 26.4 [37] | 39 (3) | 33.9 [43] | 13 (2) | 23.1 [15] |
| Any hyperglycaemia/new onset diabetes mellitus (SMQ) event | 90 (4) | 47.8 [109] | 67 (3) | 34.8 [75] | 48 (4) | 51.3 [53] | 60 (4) | 46.4 [65] | 50 (3) | 44.2 [56] | 24 (3) | 38.6 [25] |
| Any asthma/bronchospasm (SMQ) event | 19 (<1) | 8.3 [19] | 20 (<1) | 9.7 [21] | 5 (<1) | 4.8 [5] | 8 (<1) | 6.4 [9] | 14 (<1) | 11.0 [14] | 11 (1) | 17.0 [11] |

^a^Occurring in ≥1% of patients.

Note: n=Number of patients, #=Number of events. Note: Rate is event rate per 1000 patient-years, calculated as the number of events x 1000, divided by the total duration at risk.

AESI, adverse event of special interest; BMD, bone mineral density; CMH, chronic mucus hypersecretion; CNS, central nervous system; CV, cardiovascular; FF, fluticasone furoate; LRTI, lower respiratory tract infection; SMQ, Standardized MedDRA Query; UMEC, umeclidinium; VI, vilanterol.
